# Risk factors for postoperative nausea and vomiting after the removal of impacted third molars: a cross-sectional study

**DOI:** 10.1186/s12903-021-01481-8

**Published:** 2021-03-16

**Authors:** Hiroaki Hasegawa, Atsushi Abe, Hiroki Hayashi, Hiroshi Furuta, Takanori Ishihama

**Affiliations:** Department of Oral and Maxillofacial Surgery, Nagoya Ekisaikai Hospital, 4-66 Syounen-cho Nakagawa-ku, Nagoya, 454-8502 Japan

**Keywords:** Postoperative nausea and vomiting, Anesthesia, Volatile anesthetics, Total intravenous anesthesia

## Abstract

**Background:**

A better understanding of the risk factors for postoperative nausea and vomiting (PONV) could improve patient outcomes. This study aimed to analyze the risk factors for PONV and its onset after third molar impaction surgery, and to demonstrate the importance of controlling anesthesia-related factors regardless of patient-specific factors.

**Methods:**

We included patients who reported nausea and vomiting within 12 h of extubation. Patients with incomplete data, those who could not communicate, and those with gastrointestinal disorders were excluded. We evaluated patient-specific risk factors, and the use of volatile anesthetics and intraoperative fentanyl anesthetic-related factors. Multiple logistic regression analysis was performed taking patient background factors into account.

**Results:**

In total, 182 patients who underwent disimpaction of the third molar under general anesthesia between January 2017 and December 2018 at Nagoya Ekisaikai Hospital, were included. Approximately 12.6% (n = 23) patients experienced PONV, with no significant difference in terms of sex, smoking status, age, and body mass index compared to patients without PONV. Multiple logistic regression analysis revealed no interaction between fentanyl and volatile anesthetics. The major risk factor for PONV was the use of volatile anesthetics. Patients in whom anesthesia was maintained by volatile anesthetics were 13.35 times more likely to have PONV than those in whom total intravenous anesthesia was induced (*P* < 0.001).

**Conclusion:**

Maintenance of anesthesia with volatile anesthetics is a risk factor for PONV.

## Background

Postoperative nausea and vomiting (PONV) is a frequently occurring complication, with a general incidence of 20–30% [1, 2]. Moreover, it is one of the major complications after oral and maxillofacial surgery, with an incidence of 17.5–44.7% [3, 4]. PONV inflicts unexpected pain on the patient. In addition, it adds to expenses for medicine, nursing care, long-term hospital stays, and re-hospitalizations [5].

Many guidelines recommend assessing patients’ risk for PONV and taking precautions based on the risk factors [6]. Presently, the measures taken to prevent PONV mainly involve the use of medications such as antiemetics. Although the risk of PONV is generally low, we consider it to be an ongoing problem as its incidence has not decreased in recent years [7].

Although there are many reports addressing the onset of PONV after mandibular osteotomy and oral and maxillofacial trauma and tumor surgery, there have been few studies on PONV following extraction of impacted third molars [3, 4]. The purpose of this study was to analyze the risk factors for PONV and to demonstrate the importance of controlling anesthesia-related factors regardless of the patient-specific factors. Therefore, we examined the risk factors for PONV after extraction of the impacted third molar under general anesthesia.

## Methods

### Patients and data extraction

Patients with incomplete data, those who could not communicate, those with gastrointestinal disorders, and those with previous surgery under general anesthesia were excluded from this study. The Apfel score was used to conduct a PONV risk assessment. Anesthesiologists oversaw the complete procedure from anesthetic induction to recovery. PONV that developed within 12 h of extraction was determined from the medical record description by nurses and doctors.

### Apfel score

The simplified Apfel score considers the patient’s sex, history of PONV and/or motion sickness, smoking status, and postoperative use of opioids. One point is added for each applicable item, with a maximum score of 4 points. In accordance with the guidelines, Apfel scores were divided into Low (0–1), Mild (2), and High (3–4).

### Ethics

This cross-sectional study was approved by the Nagoya Ekisaikai Hospital Ethics Committee (approval number 2019-018). The study was conducted in accordance with the Strengthening the Reporting of Observational Studies in Epidemiology (STROBE) guidelines for reporting observational studies.

### Induction of general anesthesia

Induction was performed using propofol (1–2 mg/kg), remifentanil (0.2–0.5 μg/kg/min), and rocuronium (0.6–0.9 mg/kg), followed by nasal tracheal intubation. Anesthesia was maintained while the patient received oxygen (at a flow rate of 1.0–2.0 L/min) and air inhalation. Desflurane 3.0–4.0% and remifentanil 0.2–0.3 μg/kg/min were continuously administered and adjusted according to the circulatory dynamics.

Induction was performed using propofol target-controlled infusion (3–5 μg/mL), remifentanil (0.2–0.5 μg/kg/min), and rocuronium (0.6–0.9 mg/kg), finally followed by nasotracheal intubation. Anesthesia was maintained during oxygen delivery (at a flow rate of 1.0–2.0 L/min) and air inhalation. Propofol was continuously administered with a target blood concentration above 1 μg/mL. Remifentanil 0.2–0.3 μg/kg/min was continuously administered and adjusted according to the circulatory dynamics. Fentanyl 2 μg/kg was administered prior to remifentanil administration. After surgery, 2 mg/kg sugammadex was administered intravenously and stopped at the time of awakening. Muscle relaxation during this time was determined to be sufficient. PONV onset was then analyzed as a function of sex, age, body mass index (BMI), and anesthesia.

### Statistical analysis

Normality of the data was assessed using the Shapiro–Wilk test. Spearman’s correlation coefficient (Spearman’s delta) was used to assess the relationship between each factor (single correlation analysis) assuming nonparametric data. Anesthesia-related factors were classified based on the presence or absence of inhalation and use of fentanyl and compared to patient background factors (age, sex, BMI, and smoking status). Age and BMI were tested using the Mann–Whitney U test assuming nonparametric variability. Sex and smoking status were tested using Fisher’s exact test. The two-tailed significance level of the statistical tests was set at 0.05. Factors affecting PONV onset were the target variables, while the use of inhalation and fentanyl were the main factors. Logistic regression analysis was performed with age, sex, BMI, and smoking status as explanatory variables. The objective variable was the presence or absence of PONV. Logistic regression analysis was performed with use of fentanyl, use of volatile anesthetic, age, sex, BMI, and smoking status as explanatory variables. The analysis was performed with forced selection of explanatory variables. The Wald test was applied to test for the regression coefficient. All statistical analyses were performed using IBM SPSS Statistics 24.0 for Windows (IBM Corp., Chicago, IL, USA).

## Results

A cross-sectional study was conducted to assess the presence or absence of PONV in 182 patients who underwent extraction of the impacted third molar under general anesthesia between January 2017 and December 2018, at Nagoya Ekisaikai Hospital. General anesthesia was induced and maintained with volatile anesthetics in 50 patients. Total intravenous anesthesia (TIVA) was induced in a total of 132 patients.

PONV was observed in 23 of 182 cases (an incidence of 12.6%). Detailed information about patient characteristics are shown in Table 1. Table 2 classifies the risk factors for PONV (female, non-smoker, postoperative opioids, history of PONV/motion sickness) in terms of the Apfel Score. The higher the total score, the higher the risk of PONV. There was no correlation between PONV and the Apfel score. There was no significant difference in sex, smoking status, age, and BMI.

**Table 1:** Patient characteristics

Factor	Group	PONV (+) (n = 23)	PONV (−) (n = 159)	*P* value
Sex (%)	Male	12 (17.1)	58 (82.9)	0.172
	Female	11 (9.8)	101 (90.2)	
Smoking (%)	Yes	1 (6.7)	14 (93.3)	0.697
	No	22 (13.2)	145 (86.8)	
Age (years)		24 (16–83)	25 (16–68)	0.919
BMI (Kg m^−2^)		21 (16–27)	22 (16–35)	0.239

*PONV* postoperative nausea and vomiting, *BMI* body mass index

**Table 2:** Apfel scores

Score	Group	PONV (+) (n = 23)	PONV (−) (n = 159)
0–1	Low	13 (16.7%)	55 (83.3)
2	Mild	10 (9.6%)	104 (90.4)
3–4	High	0 (0%)	0 (0%)

*PONV* postoperative nausea and vomiting

Anesthesia-related factors are shown in Table 3. There were significant differences observed with the use of fentanyl and the use of volatile anesthetics. PONV was observed in 36% (n = 18) of the cases in which anesthesia was maintained with volatile anesthetics compared to 3.8% (n = 5) of those in which TIVA was induced. The use of volatile anesthetics increased the incidence of PONV significantly (*P* < 0.001). Moreover, the use of perioperative opioids increased the incidence of PONV significantly (*P* < 0.001). Analysis of factors affecting PONV is shown in Table 4.

**Table 3:** Anesthesia-related factors

Factor	Group	PONV (+) (n = 23)	PONV (−) (n = 159)	*P* value
Type of anesthesia	Inhalation	18 (36.0)	32 (64.0)	
	TIVA	5 (3.8)	127 (96.2)	< 0.001
Perioperative opioids	Yes	14 (27.0)	38 (73.0)	
	No	9 (6.9)	121 (93.1)	< 0.001

*PONV* postoperative nausea and vomiting, *TIVA* total intravenous anesthesia

**Table 4:** Multivariate analysis of factors affecting PONV

Variable	Odds ratio	95% CI	*P* value
Type of anesthesia (inhalation)	13.35	3.89–45.84	*P* < 0.001
Perioperative opioids (yes)	1.94	0.65–5.77	0.236
Sex (female)	1.01	0.32–3.07	0.991
Smoking (no)	0.4	0.26–23.48	0.432
Age (years)	1.01	0.97–1.06	0.527
BMI (Kg m^−2^)	0.84	0.71–1.01	0.057

*PONV* postoperative nausea and vomiting, *BMI* body mass index

There was no confounding by patient background factors. Multiple logistic regression analysis showed that the use of inhalation was a significant factor for PONV onset. The probability of developing PONV was 13.35 times higher in case of inhalation compared to no inhalation (*P* < 0.001).

## Discussion

In this study, 23 of the 182 patients judged to have Low and Mild (12.6%) Apfel scores developed PONV. Under general anesthesia, patients undergoing extraction of impacted third molars are less likely to develop PONV than those undergoing procedures such as cholecystectomy, laparoscopic surgery, and gynecological surgery. This has been attributed to lesser bleeding, a shorter operation time, and fewer perioperative medications.

Previously identified risk factors for PONV onset include age, sex, PONV history, motion sickness, smoking status, postoperative opioid use, and duration and type of surgery. Women are more likely to develop PONV (odds ratio [OR] of 2.57; 95% confidence interval [95% CI] 2.3–2.8) compared to men [1], and non-smokers are more likely to develop PONV compared to smokers [1]. A history of motion sickness or PONV has been identified as a late-onset PONV risk factor [1].

In this study, regardless of sex, age, BMI, smoking status, and intraoperative fentanyl use, the use of volatile anesthetics increased the risk for PONV 13.35 times compared to intravenous anesthetic induction with propofol. Volatile anesthetics have been identified as a risk factor for PONV that may develop within 2 h of surgery, with a high OR of 1.82 [8–10]. There is no significant difference based on the type of volatile anesthetic used such as sevoflurane or desflurane [11]. In contrast, TIVA by propofol has been shown to prevent early PONV within 6 h of surgery and reduce the risk of PONV by approximately 25% [12]. Hammas et al. [13] verified the effect of propofol on nausea and vomiting caused by ipecacuanha, which causes serotonin release. They observed that propofol antagonizes serotonin, which is one of the reasons propofol is said to have antiemetic effects. However, there is little evidence to support this claim, although there are many reports showing that avoiding volatile inhalation anesthesia reduces PONV significantly [9, 10, 14].

Postoperative pain may also induce PONV and avoiding narcotic analgesics to prevent PONV may not necessarily lead to prevention of PONV. In addition, the OR of PONV onset is reported to be 1.39 when narcotic analgesics are used after surgery [1].

In this study, the use of fentanyl was not directly related to the onset of PONV. For intraoperative use, this is not a significant risk factor, and in randomized controlled trials of more than 5000 patients, the PONV onset has been reported not to decrease even if short-acting remifentanil is used instead of fentanyl [9]. In addition, when a combination of local anesthetics is used for extraction of impacted third molars, fentanyl is usually not required after surgery. In this study, fentanyl was used for intubation analgesia (approximately 50–100 μg), and postoperative pain was addressed in all cases with the use of non-steroidal anti-inflammatory drugs or acetaminophen. For this reason, the use of fentanyl during surgery was treated as an independent variable, and the total usage of fentanyl and remifentanil was not analyzed. In the present study, there was no interaction between the use of volatile anesthetics and the use of fentanyl. It was found that the main risk factor for PONV was the use of volatile anesthetics. Therefore, we consider that TIVA should be selected over inhalational anesthesia where possible, even when the risk of developing PONV is low. In addition, using TIVA enables the reduction of medical costs, leading to improved patient satisfaction.

The accuracy of evaluation by the Apfel score is calculated from the Receiver Operating Characteristic (ROC) curve; we do not consider the accuracy to be sufficient as the area under the ROC curve is approximately 0.68–0.77 [15]. In addition, there are many factors with insufficient evidence to be considered true risk factors of PONV, including racial differences, drug administration timings, differences among facilities, and approach of the medical department. Based on the guidelines [6], a low Apfel score does not necessitate prevention. A Mild Apfel score justifies administration of 1–2 different drugs, while a High Apfel score justifies a combination of 2–3 drugs (e.g. ondansetron + dexamethasone + TIVA) [6].

The Apfel score has many invariant elements and is difficult to control. No specific measures are recommended for the low-risk group of PONV, although it is still likely that PONV will occur. However, antiemetic drugs have potential side effects, and it is better to avoid aggressive antiemetic therapy where the risk of PONV is low. Thus, understanding and avoiding controllable risk factors provides an attractive alternative for preventing possible PONV. There are many risk factors for PONV; however, anesthetic-related factors are of particular interest as they can be controlled by the operator.

In addition to the variables measured in this study, there is a possibility that there are other variables confounding the use of volatile anesthetics and fentanyl. The method of anesthetic maintenance is selected by the anesthesiologist based on the Apfel score. Thus, there may be an inherent bias in selecting TIVA with propofol over volatile anesthetics for maintenance of anesthesia. Since bispectral index monitoring was not implemented in all cases, we could not control the anesthetic depth analysis, and the results may therefore lack objectivity in this regard.

We secured the sample size (n = 182) that was required for statistical analysis. However, a multivariate analysis with few cases (n = 23) reduces the statistical power. In future, further prospective studies are needed, with the above considerations taken into account.

## Conclusions

Even when the risk of developing PONV is low, TIVA has a lower risk compared to volatile anesthetics and may therefore be a better option.

## Data Availability

The raw data are confidential and cannot be readily shared. Researchers need to obtain permission from the institutional Review Board and apply for data access to The Ethics Committee of Nagoya Ekisaikai Hospital.

## Abbreviations

PONV: Postoperative nausea and vomiting
TIVA: Total intravenous anesthesia
STROBE: Strengthening the Reporting of Observational Studies in Epidemiology
BMI: Body mass index
OR: Odds ratio
CI: Confidence interval
ROC: Receiver Operating Characteristic
